# Inferring microRNA and transcription factor regulatory networks in heterogeneous data

**DOI:** 10.1186/1471-2105-14-92

**Published:** 2013-03-11

**Authors:** Thuc D Le, Lin Liu, Bing Liu, Anna Tsykin, Gregory J Goodall, Kenji Satou, Jiuyong Li

**Affiliations:** 1 School of Information Technology and Mathematical Sciences, University of South Australia, Mawson Lakes, SA, 5095, Australia; 2 Children’s Cancer Institute Australia, Randwick NSW, 2301, Australia; 3 Centre for Cancer Biology, SA Pathology, Adelaide, SA, 5000, Australia; 4 School of Molecular and Biomedical Science, University of Adelaide, Adelaide, SA, 5005, Australia; 5 Department of Medicine, University of Adelaide, Adelaide, SA, 5005, Australia; 6 Kanazawa University, School of Natural Science and Technology, Kanazawa, Japan

## Abstract

**Background:**

Transcription factors (TFs) and microRNAs (miRNAs) are primary metazoan gene regulators. Regulatory mechanisms of the two main regulators are of great interest to biologists and may provide insights into the causes of diseases. However, the interplay between miRNAs and TFs in a regulatory network still remains unearthed. Currently, it is very difficult to study the regulatory mechanisms that involve both miRNAs and TFs in a biological lab. Even at data level, a network involving miRNAs, TFs and genes will be too complicated to achieve. Previous research has been mostly directed at inferring either miRNA or TF regulatory networks from data. However, networks involving a single type of regulator may not fully reveal the complex gene regulatory mechanisms, for instance, the way in which a TF indirectly regulates a gene via a miRNA.

**Results:**

We propose a framework to learn from heterogeneous data the three-component regulatory networks, with the presence of miRNAs, TFs, and mRNAs. This method firstly utilises Bayesian network structure learning to construct a regulatory network from multiple sources of data: gene expression profiles of miRNAs, TFs and mRNAs, target information based on sequence data, and sample categories. Then, in order to produce more meaningful results for further biological experimentation and research, the method searches the learnt network to identify the interplay between miRNAs and TFs and applies a network motif finding algorithm to further infer the network.

We apply the proposed framework to the data sets of epithelial-to-mesenchymal transition (EMT). The results elucidate the complex gene regulatory mechanism for EMT which involves both TFs and miRNAs. Several discovered interactions and molecular functions have been confirmed by literature. In addition, many other discovered interactions and bio-markers are of high statistical significance and thus can be good candidates for validation by experiments. Moreover, the results generated by our method are compact, involving a small number of interactions which have been proved highly relevant to EMT.

**Conclusions:**

We have designed a framework to infer gene regulatory networks involving both TFs and miRNAs from multiple sources of data, including gene expression data, target information, and sample categories. Results on the EMT data sets have shown that the proposed approach is able to produce compact and meaningful gene regulatory networks that are highly relevant to the biological conditions of the data sets. This framework has the potential for application to other heterogeneous datasets to reveal the complex gene regulatory relationships.

## Background

The regulation of gene expression is a critical mechanism in the control of biological processes in cellular organisms. At the transcriptional level, the main regulators contributing to the control are transcription factors (TFs), proteins that bind to cis-regulatory elements in the gene promoter regions [1]. By activating or repressing their target genes, TFs can regulate the global gene expression program of a living cell, and form transcriptional regulatory networks [2-4].

Recent studies have identified that microRNAs (miRNAs) play an important role in gene regulation at the post-transcriptional level. The regulation process takes place via mRNA cleavage or translational repression, with miRNAs binding to the 3’-untranslated regions (3’-UTRs) of target mRNAs through base pairing to complementary sequences [5-8]. It has also been demonstrated in a body of literature that miRNAs regulate a wide range of biological processes in proliferation [9,10], metabolism [11,12], differentiation [13], development [14,15], apoptosis [12,16,17], cellular signaling [18] and even cancer development and progression [7,19,20].

It is necessary to draw a unified picture for the regulatory relationships between TFs, miRNAs and genes. However, a challenge is that the combined regulations of miRNAs and TFs are complicated, since they involve not only the interactions between each regulator and their target genes, but also the interactions between the regulators themselves. Studies of the gene regulatory networks with the presence of both TFs and miRNAs will help elucidate the regulatory mechanisms involving both direct and indirect regulatory relationships. However, It is still highly unlikely for these relationships to be discovered by biological experiments directly, as the process would be extremely costly and time consuming. On the other hand, well-designed computational approaches may facilitate the understanding of such complex relationships.

Previously, researchers studied the co-regulation of TFs and miRNAs by finding out their shared downstream targets [21,22]. The methods used probabilistic models or statistical tests to measure the significance of the shared targets between the regulators, and to remove the insignificant co-regulating interactions that occurred by chance. Gene enrichment analysis was used in [23] to identify significant co-regulation between the transcriptional and post-transcriptional layers. They found that some biological processes emerged only in co-regulation and that the disruption of co-regulation may be closely related to cancers, suggesting the importance of the co-regulation of miRNAs and TFs. In [23] available predicted targets databases are used to construct the network, and then Gene Ontology (GO) was used to discover the significant functional co-regulation pairs. Tran et al. [24] proposed a rule based method to discover the gene regulatory modules that consist of miRNAs, TFs, and their target genes based on the available predicted target binding information. Le Béchec et al. [25] integrated available target prediction databases to construct a regulatory network that involves miRNAs, TFs, and mRNAs. This work provides a good resource for exploring the regulatory relationships or identifying the network motifs. However, target prediction based on sequences have high rate of false discoveries, which affect the quality of the discoveries of the above mentioned methods. It would be ideal if expression data can be used to refine the discoveries.

Roqueiro et al. [26] proposed a method to identify the key regulators (miRNAs or TFs) of pathways. The method used Bayesian inference on known pathway structures to infer a set of regulators in the pathway network. The Bayesian network in this method was constructed manually using the known KEGG pathways by removing the cycles in the pathways and applying some filtering criteria. The method drew findings based on existing knowledge and provided a good resource for other methods to validate their results. However, it may not be good for exploratory study.

Recently, Huang et al. [27] developed a web tool (mirConnX) for constructing the regulatory networks that include miRNA, TFs, and mRNAs. The built networks can be further analysed to identify network motifs. The method has used both predicted targets and expression data to build the network. The method integrated the association network based on expression data and the prior network based on sequence data. However, an edge in this network shows association, which may not indicate a regulation relationship. A strong association of A and B may be a result of a common regulator which regulates both A and B. Zacher et al. [28] proposed a Bayesian inference method based on expression data to explain the activity of miRNAs and TFs. However, this approach does not take into account the interactions between miRNAs and TFs.

In this paper, we present a framework to construct the complex regulatory network with three components: TFs, miRNAs, and target genes. Our approach aims to discover the regulatory relationships of miRNAs and TFs on their target genes respectively, as well as the interplay between the two different types of regulators. The method utilises multiple sources of data, including gene expression data, target information of each regulator based on sequence data, and sample categories (conditions). To test the proposed method, we use the expression data from the NCI-60 panel of cell lines [29], and investigate the interactions that may involve in the biological process of epithelial-to-mesenchymal transition (EMT).

## Methods

### Notation and definitions

Consider three expression data sets profiling *K* miRNAs, *I* TFs, and *J* mRNAs across *S* samples, respectively. Let ***x***={*x*_*k*_},***y***={*y*_*i*_},***z***={*z*_*j*_} be the vectors of miRNAs, TFs, and mRNAs, respectively, where 1≤*k*≤*K*,1≤*i*≤*I*, and 1≤*j*≤*J*. Each sample is labelled by its category, i.e. the biological condition of the samples, such as cancer or normal.

**Figure 1:**
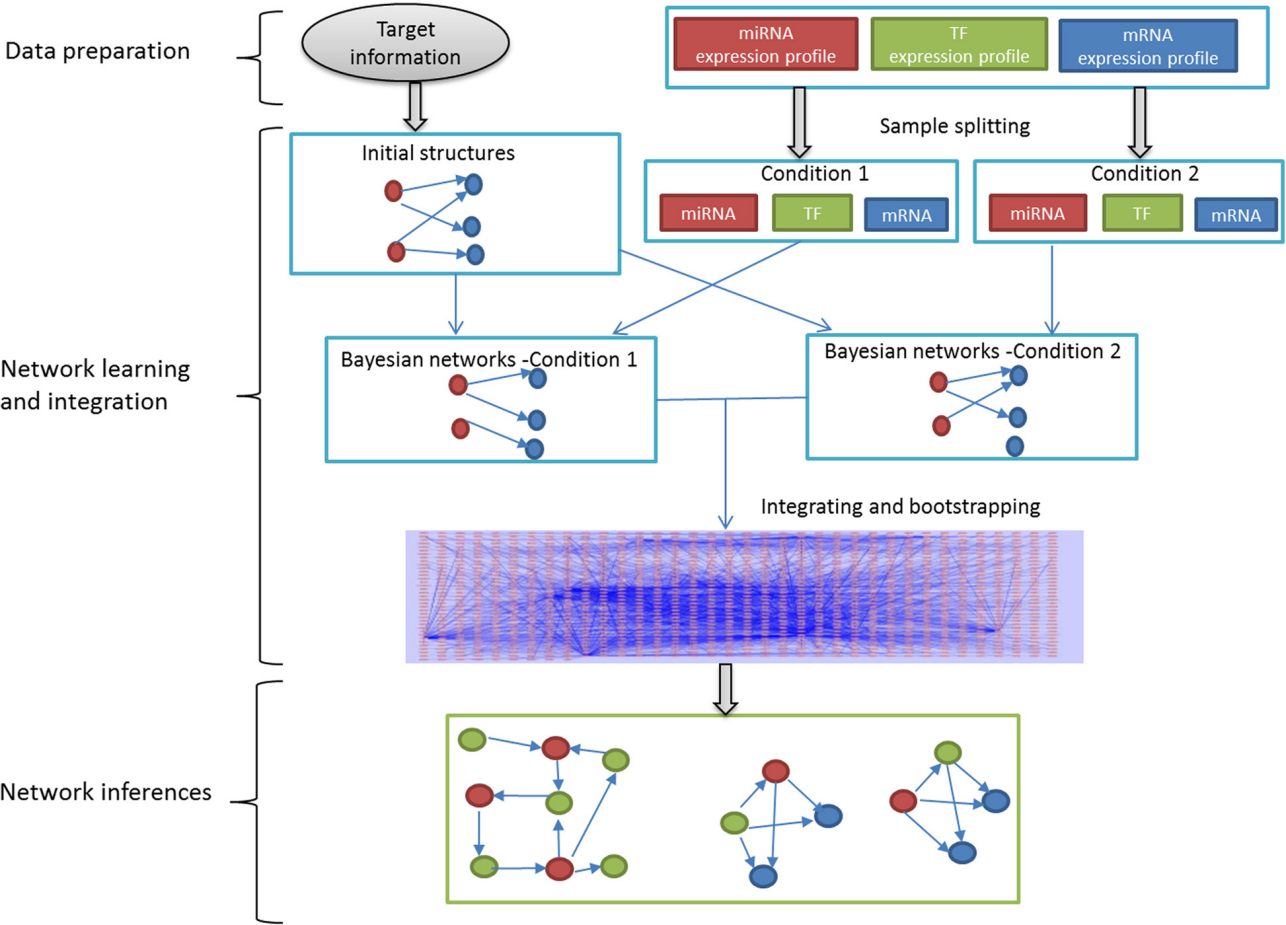
**Method overview.** The method utilises Bayesian network learning and graph search to produce a three-component regulatory network (miRNA-TF-mRNA) from multiple sources of data. Target information is used to create the initial structure representing the interactions between miRNA-mRNA, miRNA-TF, TF-miRNA, TF-TF, and TF-mRNA. For illustration, we only draw one bipartite graph in the initial structure. Expression profiles are then used in the Bayesian network learning procedure to construct the networks in each sample condition. Bootstrapping and averaging procedure is used to integrate the Bayesian networks learnt from each condition into the integrated global network. The interplays between miRNAs and TFs and the network motifs that involve at least 2 regulators are extracted from the global network and are final results.

In this paper, our goal is to discover the interactions between ***x***,***y***,***z*** (***x*** and ***y*** are regulators) supported by the expression data and under the constraint of target information (see Figure 1). *Target information* for a regulator is the interactions between the regulator and the regulated genes that are predicted based on the sequence data. We are particularly interested in the interactions between ***x*** and ***y*** (called the *interplay* of miRNAs and TFs), and *network motifs*, which are patterns of subgraphs that recur at frequencies much higher than those found in randomised networks [2].

### Method overview

In the remaining parts of the Methods section, we present our framework for constructing the regulatory network with the co-existence of both regulators, TFs and miRNAs. The method aims to produce regulatory networks including miRNAs, TFs, and genes that are relevant to diseases. The overall process is shown in Figure 1.

There are three main steps in the framework: (1) data preparation, (2) network learning and integration, and (3) network inferences. In Step (1), we prepare the input for the network structure learning, including collecting target information for TFs and miRNAs, normalising expression data, and analysing differentially expressed genes. At the beginning of Step (2), the target information is transformed into the 5 types of network sub-structures (miRNA-mRNA, miRNA-TF, TF-miRNA, TF-TF, and TF-mRNA), which are used as the initial structure for the Bayesian network learning process (refer to Figure 1). Additionally the expression datasets are split according to sample conditions. The initial structure are evaluated based on the expression profiles in each condition. The Bayesian networks learnt under each condition are integrated using a bootstrapping and averaging procedure. Therefore the result of Step (2) is an integrated global network with three components: miRNAs, TFs, and mRNAs. In the network inference step (Step (3)), we search the global network for the subgraphs that show the interplay between miRNAs and TFs, and network motifs that involve at least two regulators.

In the following, we describe each of the three steps in detail.

### Step (1): Data preparation

Epithelial-to-mesenchymal transition (EMT) is part of the process of tissue remodeling during embryonic development and wound healing [30], and during carcinogenesis [31] when cancer cells undergo a change transforming into a more invasive tumor [30,32].

After EMT induction, cells lose their epithelial features characterised by the high E-cadherin expression level, and acquire mesenchymal characteristics, including Vimentin filaments and a flattened phenotype. By expressing proteases, cells become more invasive, and they can pass through the underlying basement membrane and migrate. These are crucial steps in the multistep process of metastasis [33].

Data used in this study contain miRNA expression profiles for the NCI-60 panel of 60 cancer cell lines obtained from Sø et al. [34] (available at [http://www.ncbi.nlm.nih.gov/geo/query/acc.cgi?acc=GSE26375]). The mRNA expression profiles for NCI-60 are downloaded from ArrayExpress [http://www.ebi.ac.uk/arrayexpress], accession number E-GEOD-5720. Cell lines categorised as epithelial (11 samples) and mesenchymal (36 samples) are used in this work.

In order to identify all the TF genes in the data sets, we use the list of TF repertoire mined from [1]. This list is then used to query against the mRNA expression profiles from NCI-60 to extract TF expression profiles.

After normalising the expression data of miRNAs, TFs, and mRNAs, differentially expressed gene analysis is conducted respectively to all the three components, TFs, miRNAs, and mRNAs. The differentially expressed genes between epithelial and mesenchymal samples are identified using the *limma* package of Bioconductor [35] with the Benjamini and Hochberg’s (BH) correction method [36]. 148 probes of TFs, and 2251 probes of mRNAs are identified as differentially expressed at significant levels (adjusted *p*-value <0.1). Also 43 probes of miRNAs are identified with adjusted *p*-value <0.01. The reason for choosing adjusted 0.01 as the cut-off for miRNA differentially expressed analysis is that the *B* statistic value output from *limma* changes the value significantly between adjusted *p*-value <0.01 and adjusted *p*-value >0.01. This is a good sign for using the value of 0.01 as a cut-off, and the number of miRNAs obtained with this cut-off is also reasonable for analysing the results. (The details of differentially expressed genes are in Additional file 1).

The data input to the Bayesian network learning in the next step is the expression profiles of three components, miRNAs, TFs, and mRNAs. To integrate the data profiled from different platforms, we discretise the expression values of each gene in each sample to binary values (standing for up-regulation and down-regulation) by using the median of each array as the cut-off.

Another input to the Bayesian network learning is the target information, which is used as the prior knowledge (initial Bayesian network structure) to reduce the search space in the learning. miRNA targets and TF targets are collected via commonly used databases. These databases usually predict target genes using sequence data. In this paper, we are particularly interested in the information of TFs targeting both mRNA and miRNA genes, and the miRNAs targeting mRNA and TF genes. We use TRANSFAC 9.3 [37] and the promoter database [38] to generate TF target information. TF target information for TF-mRNA and TF-TF pairs is obtained from CRSD [39], the database utilising and integrating six well-known large scale databases, including TRANSFAC 9.3 and promoter databases. To obtain the TF-miRNA target information, we use MIR@NT@N downloaded from [25]. Meanwhile miRBase V5.0 [40] from the Bioconductor package RmiR.Hs.miRNA 2.11 is used to build the putative target for miRNAs. The detailed results of all target information are shown in the Additional file 2.

### Step (2): Bayesian networks structure learning and integration

Bayesian network structure learning has been widely used for discovering gene-gene interaction networks [41], but not often for the discoveries of the interactions between regulators and their target genes. To represent the interactions between regulators (miRNAs and TFs) and between the regulators and their target genes in a Bayesian network, regulators and target genes are denoted by nodes and regulatory interactions are denoted by directed edges. When the expression data of regulators and target genes are given, we can use Bayesian network structure learning to discover the interactions. To start the learning process, we use the target information of regulators to initialise the search space. Therefore in this step, we take the expression profiles and target information as the input to produce a network that indicates the interactions between miRNA-TF, miRNA-mRNA, TF-miRNA, TF-TF, and TF-mRNA. The following four sub-steps are carried to obtain the network.

#### Step (2.1): Sample splitting

To explore all possible interactions including up-, down-, and mix- regulations (up-regulation in one condition and down-regulation in the other) in different biological conditions, in [42] we developed the “splitting and averaging” strategy for Bayesian networks structure learning. This strategy splits samples in a data set by their categories of biological conditions. Bayesian network structure learning is used to learn the networks from the samples of each condition respectively, and these networks are then integrated or averaged into a single network. In our current problem, we firstly use this strategy to learn the networks for the epithelial and mesenchymal conditions separately, then obtain the integrated network from the networks learnt under the two conditions. So in this sub-step, we split each of the three expression datasets according to sample conditions, 11 samples in epithelial and 36 samples in mesenchymal (conditions 1 and 2 in Figure 1 respectively).

#### Step (2.2): Creating the initial structure

To learn a Bayesian network with *n* variables or nodes, the search space, if not restricted, will be all the possible networks formed with the variables. It has been shown that finding the best network from all the networks is NP-hard [43]. Therefore in this paper, we assume that the regulatory relationships between regulators and their target genes form a bipartite graph. This assumption reduces the search space significantly. Moreover, we use target information to initialise the network structure and the topology of the network structure is further constrained. Therefore, we are able to discover the optimal solutions using the exhaustive search method in the given search space. Graphically, the target information can be represented using bipartites as illustrated in Figure 1. There are 5 types of such bipartites or sub-structures, corresponding to our initial knowledge of the interactions of miRNA-TF, miRNA-mRNA, TF-TF, TF-miRNA and TF-mRNA. These bipartites are used as the initial structure for the Bayesian network learning.

#### Step (2.3): Bayesian network learning process

Each interaction in the initial structure is evaluated based on the expression data, and the high-confidence interactions are retained. The learning process searches through all possible candidate structures and evaluates the interactions with a Bayesian scoring function. The candidate structures are generated by removing edges from the initial structure one by one. The scoring function measures the degree of fitness of any candidate structure *G* to the dataset. The goal is to select the structure that best fits the data. In other words, we need to calculate the probability of each candidate structure *G* given the data *D*, *P*(*G*|*D*). According to Bayes’ theorem, we have:

$$P(G|D)=\frac{P(G)P(D|G)}{P(D)}$$

In the above formula, the term *P*(*D*) is the same for all candidate structures. Regarding the term *P*(*G*), it is quite common to assume a uniform distribution [44], and thus it is a constant. Therefore, for comparative purposes, it is sufficient to calculate only *P*(*D*|*G*) for the scoring function. In this paper, we use the BDe (Bayesian metric with Dirichlet priors and equivalent) scoring function developed by Heckerman et al. [45] in the following.

$$\begin{array}{lll} \;{\text{Score}}_{B}(D,G)=P(D|G) & =\overset{n}{\underset{i=1}{\prod }}\overset{q_{i}^{\left(G\right)}}{\underset{j=1}{\prod }}\frac{\Gamma (N_{\text{ij}}^{\left(G\right)})}{\Gamma (N_{\text{ij}}^{\left(G\right)}+M_{\text{ij}}^{\left(G\right)})}\; & \;\\ & \;\times \overset{r_{i}}{\underset{k=1}{\prod }}\frac{\Gamma (a_{\text{ijk}}^{\left(G\right)}+s_{\text{ijk}}^{\left(G\right)})}{\Gamma (a_{\text{ijk}}^{\left(G\right)})}\; & \; \end{array}$$

where: 

*n* is the number of variables (nodes), *X*_1_,*X*_2_,…,*X*_*n*_,

*q*_*i*_ is the number of different instantiations of the parent of a variable *X*_*i*_ in *G*,

*r*_*i*_ is the number of possible values of *X*_*i*_ in *G*,

$a_{\text{ijk}}^{\left(G\right)}$ are the hyperparameters for the Dirichlet prior distributions of the parameters given the network structure.

$s_{\text{ijk}}^{\left(G\right)}$ are the corresponding observations from data,

$N_{\text{ij}}^{\left(G\right)}=\underset{k}{\sum }a_{\text{ijk}}^{\left(G\right)}$, and $M_{\text{ijk}}^{\left(G\right)}=\underset{k}{\sum }s_{\text{ijk}}^{\left(G\right)}$.

More details of the Bayesian scoring function can be found in [45,46]. In practice, we use the Bayes Net toolbox for Matlab (BNT) [47], and the BDe scoring function is included in BNT. In the next step (Step (2.4)) we evaluate the confidence levels of the interactions output from the Bayesian network structure learning.

**Figure 2:**
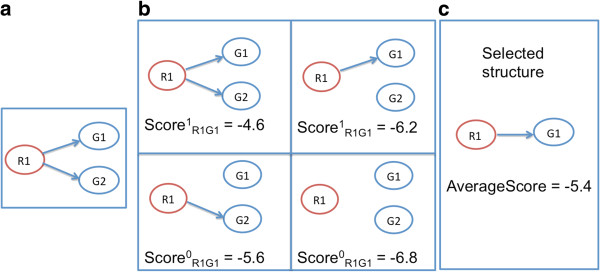
**An example of Bayesian network structure learning. (a)** The initial structure corresponding to regulator *R*_1_ and target gene *G*_1_. This initial structure is created based on target information of the regulator. **(b)** All possible candidate structures generated from the initial structure. The interaction between *R*_1_ and *G*_1_ in each candidate structure is evaluated using a scoring function. **(c)** The presence or absence of an edge between *R*_1_ and *G*_1_ based on the average score of each case. The candidate structure with higher average score will be chosen.

For illustration purpose, consider the learning procedure for the interaction between a regulator *R*_1_ and its target gene *G*_1_. Assume that in total *R*_1_ has two targets and the corresponding initial structure is in Figure 2a. The interactions in each of the four possible candidate structures (see Figure 2b) can be evaluated with the scoring function based on expression data. The scores will decide if there is no edge between *R*_1_ and *G*_1_ or an edge between them. In this example, there are two structures with an edge between *R*_1_ and *G*_1_, and two structures with no edge between them. The average score in each of the two cases is calculated and the structure with higher average score ((-4.6-6.2)/2=-5.4) is chosen (Figure 2c).

#### Step (2.4): Integrating and bootstrapping

It is common to have small number of samples in the dataset of a typical microarray experiment, which unfortunately cannot support statistically significant discoveries. To overcome this problem, bootstrapping [48] is usually used to improve the confidence of discoveries. Especially, in Bayesian network structure learning, bootstrapping can be combined with model (structure) average procedure to discover the interactions with high confidence. In this paper, the averaging procedure is firstly applied to the Bayesian network learning process across different candidate structures. This procedure is then applied to the sample splitting step across different sample conditions to calculate the average score for each interaction. Next, the score of each interaction is averaged over the number of bootstrapping, and the confidence levels are estimated based on a statistical model as illustrated in the next paragraph. The interactions with high confidence (*p*-value <0.05) are selected to form the integrated network (called *global network* in the paper)

Consider again the example about learning the interaction between *R*_1_ and *G*_1_. Let *n* be the number of bootstrapping iterations, *q*_*c*_ be the event of learning the interaction on the local data set *D*_*c*_ of the *c*^*th*^ condition (*c*∈{1,…,*C*}). As there are only two possible cases of interactions between *R*_1_ and *G*_1_, we approximate the whole learning process of the interaction between *R*_1_ and *G*_1_ as a Bernoulli experiment. We denote *q*_*c*_=1 when there is an edge between *R*_1_ and *G*_1_ (otherwise *q*_*c*_=0), and assume that *p*(*q*_*c*_=1)=*p*(*q*_*c*_=0)=0.5. *q*_*c*_ follows a binomial distribution *q*_*c*_∼*B*(*n*,*p*), as the samples drawn with replacement in the bootstrap are independent [49]. At the integration stage by averaging, the interactions from local data sets *D*_*c*_ are aggregated, and the interactions of the regulator *R*_1_ and its target *G*_1_ learnt through multiple data sets for the *C* different conditions (denoted as $Q_{R_{1}G_{1}}=\underset{c}{\sum }q_{c}$) also follows a binomial distribution $Q_{R_{1}G_{1}}∼B(\text{Cn},p)$. Adopting this statistical model, we are able to extract the learnt interactions at significant levels. The interaction that has the confidence level greater than the threshold will be included into the integrated global network. The Matlab codes for the whole process is available on request, and the results for the integrated global network is in Additional file 3.

### Step (3): Network inference

A challenging problem of Bayesian network learning is the complexity of the resulting networks. Bayesian network learning usually produces a large number of interactions, including false discoveries. In this step, we extract from the global network learnt in the previous step the interplay between miRNAs and TFs. We search the learnt global network for all of the interactions between miRNAs and TFs. The resulting interplay network will help elucidate the complex regulatory mechanism in specific biological conditions.

In addition to discovering the interplay between miRNAs and TFs, we use the network motif finding algorithm, Cyclus3D [50], to discover the network motifs that involve at least 2 regulators. Network motifs are patterns of sub-graphs that recur at frequencies much higher than those found in randomised networks [2]. The randomised networks satisfy the following criteria: 1) they have the same number of nodes as in the real network, 2) each node in a randomised network has the same number of incoming and outgoing edges as the corresponding node has in the real network, 3) the randomised networks used to calculate the significance of *n*-node subgraphs were generated to preserve the same number of appearances of all (*n*−1)-node subgraphs as in the real network. These criteria ensure the randomised networks have the similar structure to the real network, and ensure that a high-significance pattern is assigned not because it has a highly significant sub-pattern [2].

The resulting motifs can be considered as simple building blocks from which the network is composed [51], and are believed to have specific functions which play critical roles in biological network inference [52]. The results presented in the next section show that this network inferences step can produce a set of interactions and molecules which are highly relevant to the biological condition of the EMT datasets.

## Results

**Figure 3:**
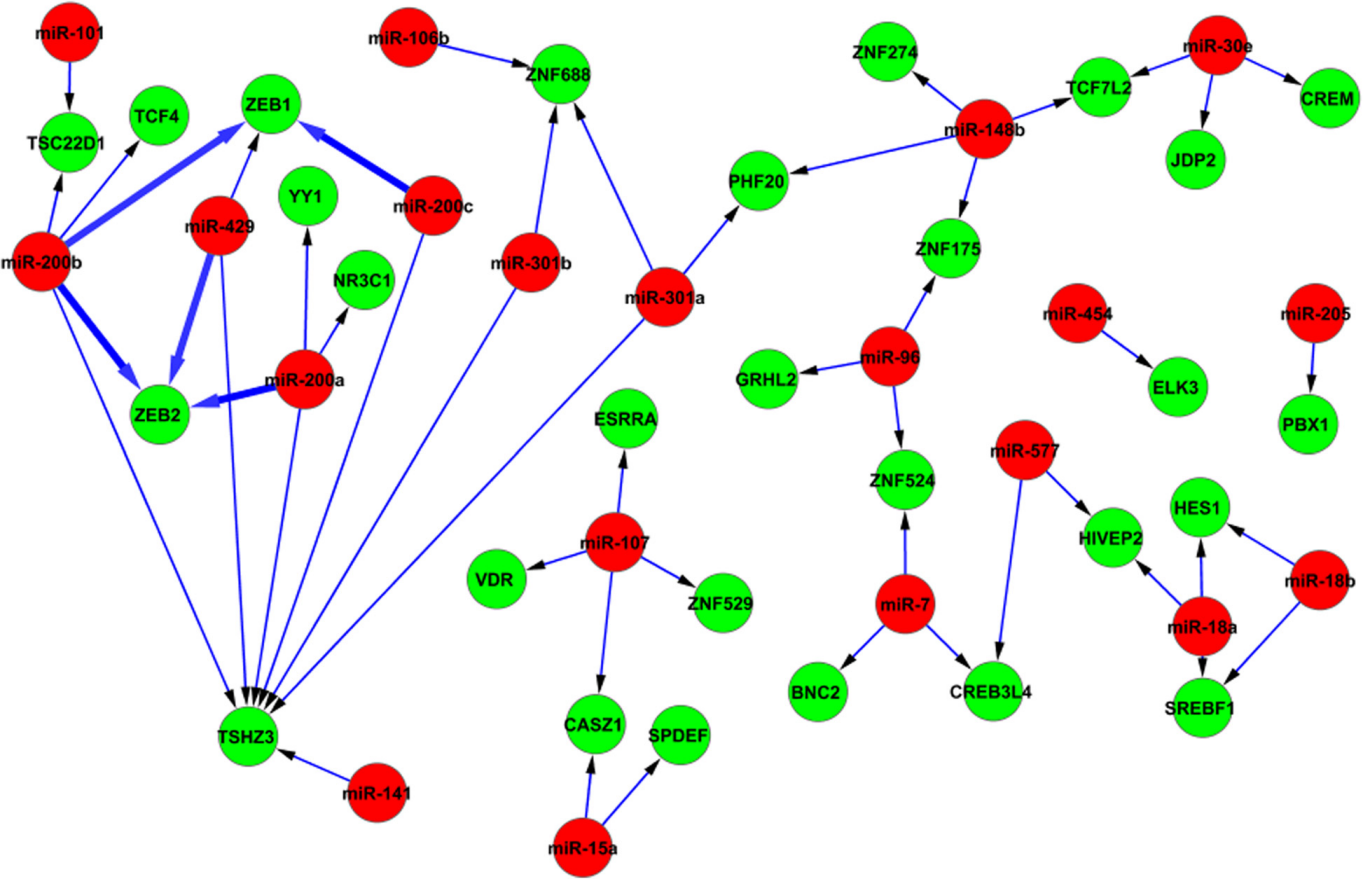
**The interplays between TFs and miRNAs - miRNAs regulate TFs.** miRNAs are coloured in red and TFs are in green. The confirmed edges are highlighted with bold lines. All of the nodes in the confirmed interactions are EMT bio-markers. They are the miR-200 family, ZEB1, ZEB2, and SNAI2 (SLUG). The miR-200 family that regulates ZEB1, ZEB2 for EMT has been confirmed by literature.

**Figure 4:**
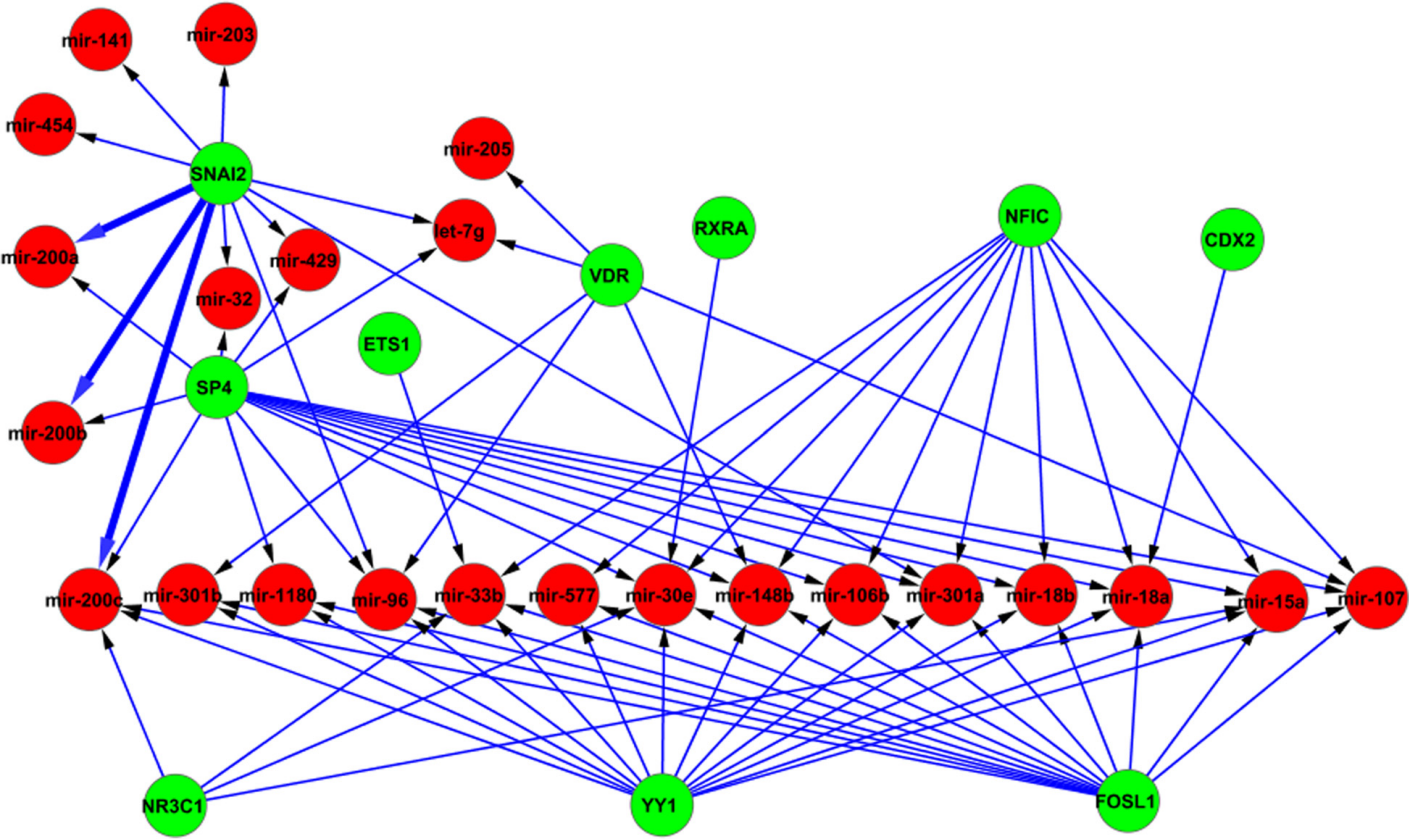
**The interplays between TFs and miRNAs - TFs regulate miRNAs.** The confirmed edges are highlighted with bold lines. All of the nodes in the confirmed interactions are EMT bio-markers. SNAI2 (SLUG) regulates miR-200 family to indirectly control ZEB1 and ZEB2 for activating EMT regulation procedure. These interactions have been confirmed by literature.

**Figure 5:**
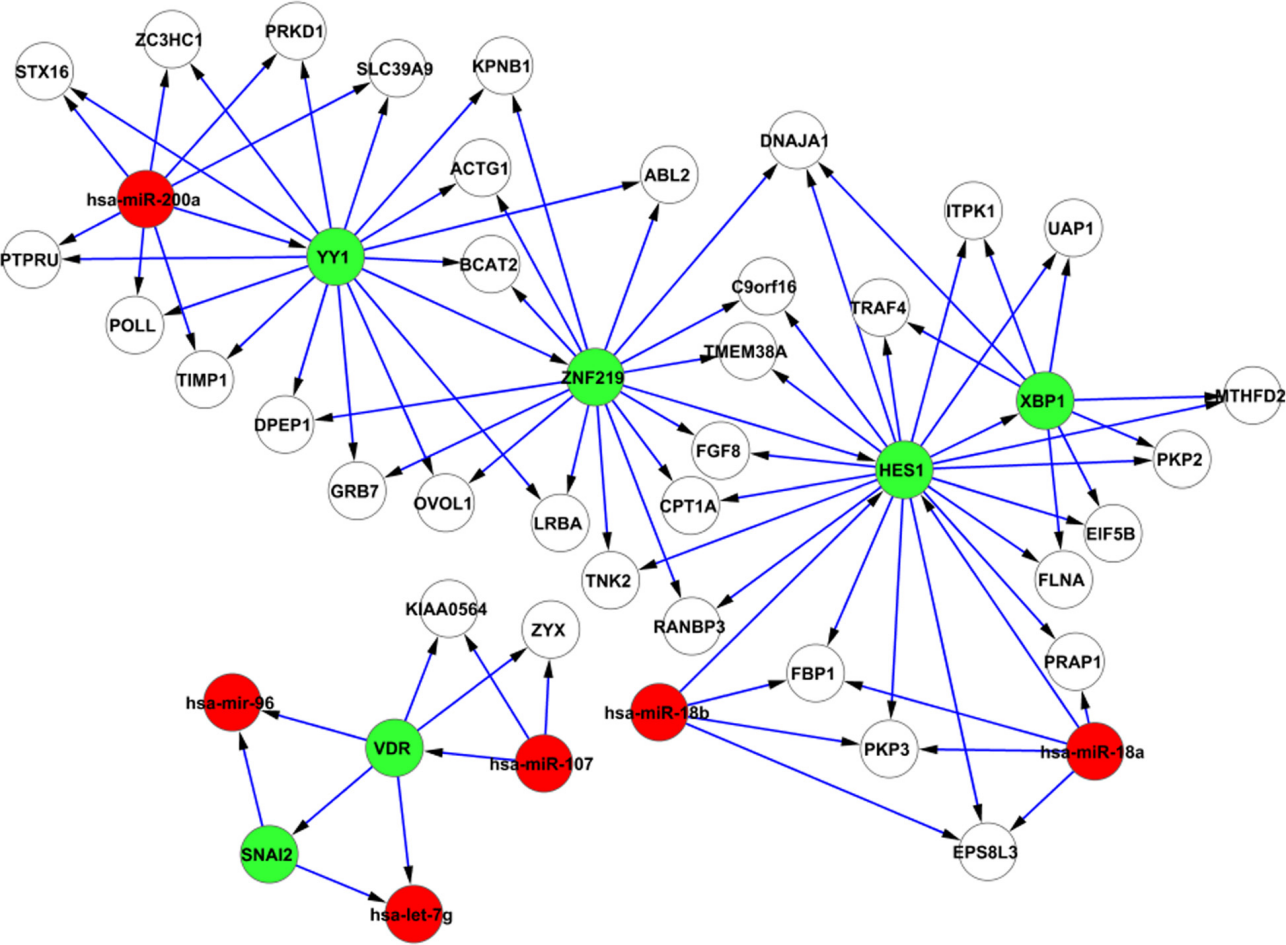
**Network motif.** Feed Forward Loop network motifs are extracted from the integrated global network. The genes identified in these network motifs are significantly enriched for biological functions of EMT.

The output of the method are two types of networks: 1) the interplays between miRNAs and TFs, with their details shown in Figure 3 and Figure 4; 2) the results of network motif finding, which are the Feed Forward Loops (FFLs) that involve at least two regulators (see Figure 5).

From Figures 3, 4, and 5, we can see that the results generated by our method are compact with only a small number of interactions. These interactions have been shown to be highly relevant to the biological conditions of EMT, and also several EMT bio-markers which have been confirmed by literature are identified by our method. In the rest of this section, firstly we present the interactions and bio-markers that have been confirmed from literature, then we describe the enrichment analysis we have conducted to show the relevance of identified genes to EMT.

### Confirmed interactions and bio-markers for EMT

**Figure 6:**
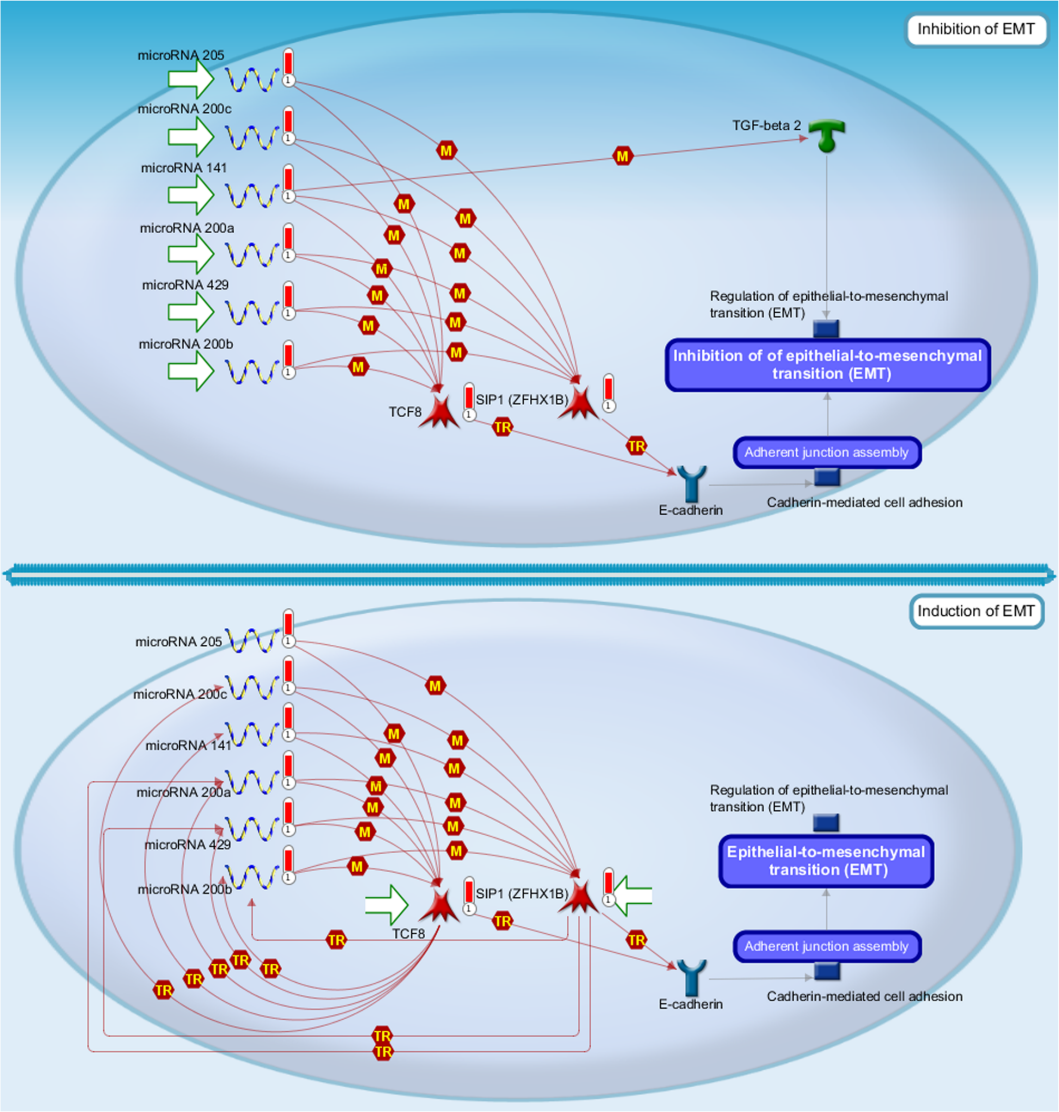
**The pathway of development_microRNA-dependent inhibition of EMT.** Genes identified by our method are marked with red bars. miR-200 family regulates ZEB1 and ZEB2 in the process of inhibition and induction of EMT. These interactions are also identified by our method.

Previous studies [33,53,54] have demonstrated that the miR-200 family targets the E-cadherin transcriptional repressors zinc finger E-box binding homeobox 1(ZEB1) and ZEB2 for EMT. These results have confirmed the interactions found using our method (shown in Figure 3), where we see that the hsa-miR-200 family (miR-200a, miR-200b, miR-200c, miR-429) regulates both ZEB1 and ZEB2. These interactions are the important interactions that are involved in the process of inhibition and induction of EMT. Figure 6 shows the process in detail where genes identified by our method are marked with red bars.

Apart from the miR-200 family, several important transcription factors that act as the bio-markers for EMT are also confirmed by our method. The two transcription factors, ZEB1 and ZEB2, which are regulated by the miR-200 family, are the markers in all three subtypes of EMT [55]. Another transcription factor that plays a crucial role in EMT is SNAI2 (SLUG). In fact, all known EMT events during development, cancer, and fibrosis appear to be associated with SNAI activation [56]. Our results suggest that SNAI2 indirectly regulates ZEB1 and ZEB2 by regulating the miR-200 family transcript (Figure 4), and in turn the miR-200 family regulates ZEB1 and ZEB2 (see Figure 3). This result is consistent with the literature as SNAI2 is confirmed to regulate the miR-200 family [57]. The other EMT bio-marker identified by our method is ETS1 (see Figure 4). It has been suggested that ETS1 is an upstream regulator of ZEB1 and ZEB2 [58], and therefore plays a critical role in activating the regulation of EMT.

### Functions of identified genes being highly enriched for EMT

The functions of the identified genes (in Figures 3, 4, and 5) and the pathways which the genes potentially constitute are analysed using GeneGo Metacore from GeneGo Inc. and the Ingenuity Pathway Analysis (IPA, Ingenuity Systems, www.ingenuity.com). The genes identified as a result of the network inference step are significantly enriched for several biological functions. The top functions output from IPA that are known to be critical for EMT are gene expression, cellular development, cellular growth and proliferation, cellular movement, and cell death. Moreover, several genes belong to the classes of invasion and migration. These classes are sub-categories of cellular movement, and they have been confirmed as the functional markers of EMT [59]. This suggests that many target genes and their interactive regulators are involved in EMT. Table 1 shows the genes in the class of invasion and migration together with their *p*-values.

**Table 1:** Identified genes are significantly involved in the functional markers of EMT

**Functions**	**Molecules**	**Number**	**p-value**
Invasion	VDR, FGF8, miR-17-5p,	16	1.26E-06 - 5.4E-03
	miR-200a-3p, miR-429, miR-7-5p		
	TIMP1, CDX2, ETS1, FLNA,		
	FOSL1, SPDEF, TNK2, YY1		
	ZEB1, ZEB2.		
Migration	ESRRA, ETS1, FOSL1,	24	3.59E-09 - 7.96E-03
	PRKD1, SNAI2, SPDEF, TIMP1,		
	TNK2, ABL2, CDX2, FGF8,		
	FLNA, GRB7, let-7a-5p, miR-16-5p,		
	miR-17-5p, miR-200a-3p, miR-429,		
	NFIC, PRAP1, PTPRU, RXRA,		
	SREBF1, ZEB1		

A significant number of genes identified in the inference step belong to the class of invasion and migration which are EMT functional markers. The results are generated by IPA.

In addition, the pathways which the genes in our results potentially constitute are identified using GeneGo Metacore. The statistically mapped pathways show that they are highly relevant to EMT. There are direct pathways that regulate EMT, such as the pathway of development_microRNA-dependent inhibition of EMT. This pathway shows the regulation of the miR-200 family and other miRNAs on the EMT bio-markers ZEB1 and ZEB2, and results in the inhibition and induction of EMT. Figure 6 shows the details of this pathway. Other direct pathways such as the development_TGF-beta-dependent induction of EMT via SMADs, and cell adhesion_tight junctions, are known to play critical roles in the regulatory procedure of EMT. The summary of these pathways and the corresponding *p*-values are given in Table 2.

**Table 2:** The statistically mapped pathways for EMT involve identified genes

***#***	**Pathway**	**p-value**
1	Development_microRNA-dependent inhibition of EMT	8.645E-17
2	Development_WNT signaling pathway. Part 2	2.227E-05
3	Development_TGF-beta-dependent induction of EMT via SMADs	7.325E-05
4	Cell adhesion_Chemokines and adhesion	4.632E-04
5	Cell adhesion_Tight junctions	1.614E-03
6	Cell adhesion_Role of tetraspanins in the integrin-mediated cell adhesion	1.748E-03
7	Development_TGF-beta receptor signaling	4.154E-03

The mapped pathways involve identified genes that are important for EMT. The results are generated by GeneGo Metacore.

## Discussion

During the past few decades, considerable efforts have been made to explore the transcriptional regulatory networks in which transcription factors play the role as a main regulator. Other recent studies have investigated the post-transcriptional regulatory networks with miRNAs as the main regulator. However, with the ultimate goal of achieving a profound understanding of the mechanisms that control gene activities, it is sensible and desirable to find regulatory relationships involving both types of regulators from diverse sources of data.

In this paper, we utilise Bayesian network learning in constructing the network, but the integrated global network in general is not a Bayesian network. For instance, one of the requirements for Bayesian networks is that the network structure must be a Directed Acyclic Graph (DAG). Our integrated global network may include some loops of interactions where two regulators interact with each other, hence it is not a Bayesian network. Such cyclic network behaviour is more reasonable in reality, as more and more feedback loops between miRNAs and TFs are being reported. For instance, the ZEB/miR-200 pair is a feedback loop that regulates EMT [60]. Therefore, the integrated global network may provide more information than normal Bayesian networks which are DAGs.

In the network inference step, we use network motif finding algorithm to discover the sub-networks that recur at statistically significant level. Interestingly, the results from these small sub-networks still retain several important interactions and molecules relevant to the biological condition of the dataset. The relationships between the significance in frequency of graphs and biological functions are still open and interesting research topics. In the dataset used in this paper, the results are supportive for this hypothesis. An advantage of motif finding is that it produces a manageable number of interactions that can be used for further experimentation. The results from this paper, therefore, can provide good resources for future validating experiments.

While the network motifs found based on the regulatory network may provide useful patterns to guide biological experiments, these motifs depend on the structure of the regulatory network. The structure of the regulatory network in this paper is obtained based on the assumption that miRNAs and TFs are regulators and mRNAs are targets. However, the knowledge of gene regulatory relationships is still limited and the assumption may not always hold in reality. When the structure of the regulatory network changes the resulting network motifs may change too.

In the paper, we use the differentially expressed genes as the nodes for the gene regulatory network. We assume that genes whose expression levels do not change significantly between conditions would not play an important role in the regulatory network. There may be the case that a gene is the target of two regulators that cancel out each other, resulting in the expression level of the target gene unchanged. However, to make our method computationally practical we do not consider such cases.

To start the process of Bayesian network structure learning, target information is used to initialise the network. The target information based on sequence data may involve false discoveries. Bayesian network structure learning uses gene expression data to evaluate the confidence level of each interaction (edge) in the initial network, and only the interactions of high confidence are integrated into the global network. Therefore, graphically the set of edges in the global network is a subset of the set of edges in the initial network. The enrichment analysis shows that the important interactions for EMT are retained in the global network, demonstrating the effectiveness of the method. Other high-confidence interactions provide strong hypotheses for experimental validations.

## Conclusions

In this study, we have proposed a framework for inferring complex gene regulatory networks using diverse sources of data, including target information for regulators, expression profiles, and sample categories. The interplay between regulators and the motifs with which they regulate target genes are revealed in the three-component network, and it is impossible to infer the interplay from any single regulator regulatory networks. The analysis of the EMT datasets has produced several results that have been validated by literature, a number of statistically significant interactions between miRNAs and TFs, and novel network motifs.

## Competing interests

The authors declare that they have no competing interests.

## Authors’ contributions

TL and JYL conceived this research. TL designed and performed the experiments. BL, AT and GG provided the source of data and validated the results. LL verified the learning model. TL, LL, BL, KS and JYL drafted the manuscript. All authors read and approved the final manuscript.

## Supplementary Material

Additional file 1 **Differentially expressed miRNAs, TFs, and mRNAs. ***limma* package from Bioconductor is used to identify differentially expressed miRNAs, TFs, and mRNAs. Click here for file

Additional file 2 **Target information.** The interactions that show the TF and miRNA target information. This information is used to initialise the searching space for Bayesian network learning. Click here for file

Additional file 3 **Significant interactions.** All the statistically significant interactions for the complex three-component network. These interactions represent the regulatory relationships between miR-mRNA, miR-TF, TF-miRNA, TF-TF, and TF-mRNA. Click here for file

## References

[B1] Vaquerizas MJ Kummerfeld KS Teichmann AS Luscombe MN A census of human transcription factors: function, expression and evolution Nat Rev Genet 2009 10 4 252 263 10.1038/nrg2538 19274049

[B2] Shen-Orr SS Milo R Mangan S Alon U Network motifs in the transcriptional regulation network of Escherichia coli Nat Genet 2002 31 64 68 10.1038/ng881 11967538

[B3] Lee TI Rinaldi NJ Robert F Odom DT Bar-Joseph Z Gerber GK Hannett NM Harbison CT Thompson CM Simon I Transcriptional regulatory networks in Saccharomyces cerevisiae Science 2002 298 799 804 10.1126/science.1075090 12399584

[B4] Yu H Gerstein M Genomic analysis of the hierarchical structure of regulatory networks Proc Natl Acad Sci U S A 2006 103 14724 14731 10.1073/pnas.0508637103 17003135 PMC1595419

[B5] Berezikov E Cuppen E Plasterk RHA Approaches to microRNA discovery Nat Genet 2006 38 2 8 10.1038/ng1794 16736019

[B6] Ambros V The functions of animal microRNAs Nature 2004 431 7006 350 355 10.1038/nature02871 15372042

[B7] Bartel DP MicroRNAs: genomics, biogenesis, mechanism, and function Cell 2004 116 281 297 10.1016/S0092-8674(04)00045-5 14744438

[B8] Meister G Tuschl T Mechanisms of gene silencing by double-stranded RNA Nature 2004 431 7006 343 349 [http://www.ncbi.nlm.nih.gov/pubmed/15372041] 10.1038/nature02873 15372041

[B9] Chen JF Mandel EM Thomson JM Wu Q Callis TE Hammond SM Conlon FL Wang DZ The role of microRNA-1 and microRNA-133 in skeletal muscle proliferation and differentiation Nat Genet 2006 38 2 228 233 [http://www.ncbi.nlm.nih.gov/pubmed/16380711] 10.1038/ng1725 16380711 PMC2538576

[B10] Zhao Y Samal E Srivastava D Serum response factor regulates a muscle-specific microRNA that targets Hand2 during cardiogenesis Nature 2005 436 7048 214 220 [http://www.ncbi.nlm.nih.gov/pubmed/15951802] 10.1038/nature03817 15951802

[B11] Poy MN Eliasson L Krutzfeldt J Kuwajima S Ma X Macdonald PE Pfeffer S Tuschl T Rajewsky N Rorsman P Stoffel M A pancreatic islet-specific microRNA regulates insulin secretion Nature 2004 432 7014 226 30 [http://www.ncbi.nlm.nih.gov/pubmed/15538371] 10.1038/nature03076 15538371

[B12] Xu P Vernooy SY Guo M Hay BA The drosophila MicroRNA Mir-14 suppresses cell death and is required for normal fat metabolism Curr Biol 2003 13 2 790 795 12725740 10.1016/s0960-9822(03)00250-1

[B13] Esquela-Kerscher A Slack FJ Oncomirs - microRNAs with a role in cancer Nat Rev Cancer 2006 6 4 259 269 [http://www.ncbi.nlm.nih.gov/pubmed/16557279] 16557279 10.1038/nrc1840

[B14] Jin P Zarnescu DC Ceman S Nakamoto M Mowrey J Jongens Ta Nelson DL Moses K Warren ST Biochemical and genetic interaction between the fragile X mental retardation protein and the microRNA pathway Nat Neurosci 2004 7 2 113 117 [http://www.ncbi.nlm.nih.gov/pubmed/14703574] 10.1038/nn1174 14703574

[B15] Zhao Y Ransom JF Li A Vedantham V von Drehle M Muth AN Tsuchihashi T McManus MT Schwartz RJ Srivastava D Dysregulation of cardiogenesis, cardiac conduction, and cell cycle in mice lacking miRNA-1-2 Cell 2007 129 2 303 317 [http://www.ncbi.nlm.nih.gov/pubmed/17397913] 10.1016/j.cell.2007.03.030 17397913

[B16] Xu C Lu Y Pan Z Chu W Luo X Lin H Xiao J Shan H Wang Z Yang B The muscle-specific microRNAs miR-1 and miR-133 produce opposing effects on apoptosis by targeting HSP60, HSP70 and caspase-9 in cardiomyocytes J Cell Sci 2007 120 Pt 17 3045 3052 [http://www.ncbi.nlm.nih.gov/pubmed/17715156] 17715156 10.1242/jcs.010728

[B17] Xu P Guo M Hay Ba MicroRNAs and the regulation of cell death Trends Genet: TIG 2004 20 12 617 624 [http://www.ncbi.nlm.nih.gov/pubmed/15522457] 10.1016/j.tig.2004.09.010 15522457

[B18] Cui Q Yu Z Purisima EO Wang E Principles of microRNA regulation of a human cellular signaling network Mol Syst Biol 2006 2 46 [http://www.pubmedcentral.nih.gov/articlerender.fcgi?artid=1681519&tool=pmcentrez&rendertype=abstract] 16969338 10.1038/msb4100089 PMC1681519

[B19] Kim VN Nam JW Genomics of microRNA Trends Genet 2006 22 165 173 10.1016/j.tig.2006.01.003 16446010

[B20] Flynt AS Lai EC Biological principles of microRNA-mediated regulation: shared themes amid diversity Nat Rev Genet 2008 9 831 842 18852696 10.1038/nrg2455 PMC2729318

[B21] Shalgi R Lieber D Oren M Pilpel Y Global and local architecture of the mammalian microRNA-transcription factor regulatory network PLoS Comput Biol 2007 3 e131 10.1371/journal.pcbi.0030131 17630826 PMC1914371

[B22] Zhou Y Ferguson J Chang JT Kluger Y Inter- and intra-combinatorial regulation by transcription factors and microRNAs BMC Genomics 2007 8 396 10.1186/1471-2164-8-396 17971223 PMC2206040

[B23] Chen CY Chen ST Fuh CS Juan HF Huang HC Coregulation of transcription factors and microRNAs in human transcriptional regulatory network BMC Bioinformatics 2011 12 Suppl 1 S41 [http://www.biomedcentral.com/1471-2105/12/S1/S41] 10.1186/1471-2105-12-S1-S41 21342573 PMC3044298

[B24] Tran DH Satou K Ho TB Pham TH Computational discovery of miR-TF regulatory modules in human genome Bioinformation 2010 2063 8 371 377 20975901 10.6026/97320630004371 PMC2951675

[B25] Béchec AL Portales-casamar E Vetter G Moes M Zindy Pj Saumet A Arenillas D Theillet C Wasserman WW Lecellier Ch MIR @ NT @ N : a framework integrating transcription factors, microRNAs and their targets to identify sub-network motifs in a meta-regulation network model BMC Bioinformatics 2011 12 67 [http://www.biomedcentral.com/1471-2105/12/67] 10.1186/1471-2105-12-67 21375730 PMC3061897

[B26] Roqueiro D Huang L Dai Y Identifying transcription factors and microRNAs as key regulators of pathways using Bayesian inference on known pathway structures Proteome Sci 2012 10 Suppl 1 Suppl 1 S15 [http://www.pubmedcentral.nih.gov/articlerender.fcgi?artid=3380732&tool=pmcentrez&rendertype=abstract] 22759573 10.1186/1477-5956-10-S1-S15 PMC3380732

[B27] Huang GT Athanassiou C Benos PV mirConnX: condition-specific mRNA-microRNA network integrator Nucleic Acids Res 2011 39 W416 W423 [http://www.pubmedcentral.nih.gov/articlerender.fcgi?artid=3125733&tool=pmcentrez&rendertype=abstract] 10.1093/nar/gkr276 21558324 PMC3125733

[B28] Zacher B Abnaof K Gade S Younesi E Tresch A Fröhlich H Joint Bayesian inference of condition-specific miRNA and transcription factor activities from combined gene and microRNA expression data Bioinformatics (Oxford, England) 2012 28 13 1714 1720 [http://www.ncbi.nlm.nih.gov/pubmed/22563068] 10.1093/bioinformatics/bts257 22563068

[B29] Gaur A Jewell Da Liang Y Ridzon D Moore JH Chen C Ambros VR Israel Ma Characterization of microRNA expression levels and their biological correlates in human cancer cell lines Cancer Res 2007 67 6 2456 2468 [http://www.ncbi.nlm.nih.gov/pubmed/17363563] 10.1158/0008-5472.CAN-06-2698 17363563

[B30] Savagner P Leaving the neighborhood: molecular mechanisms involved during epithelial-mesenchymal transition BioEssays 2001 23 10 912 923 [http://www.ncbi.nlm.nih.gov/pubmed/11598958] 10.1002/bies.1132 11598958

[B31] Dvorak HF Tumors: wounds that do not heal. Similarities between tumor stroma generation and wound healing N Engl J Med 1986 315 26 1650 1659 10.1056/NEJM198612253152606 3537791

[B32] Fuchs I Lichtenegger W Buehler H Henrich W Stein H Kleine-Tebbe A Schaller G The prognostic significance of epithelial-mesenchymal transition in breast cancer Anticancer Res 2002 22 6A 3415 12530097

[B33] Park SM Gaur AB Lengyel E Peter ME The miR-200 family determines the epithelial phenotype of cancer cells by targeting the E-cadherin repressors ZEB1 and ZEB2 Genes Dev 2008 22 7 894 907 [http://www.pubmedcentral.nih.gov/articlerender.fcgi?artid=2279201&tool=pmcentrez&rendertype=abstract] 10.1101/gad.1640608 18381893 PMC2279201

[B34] Sø kilde R Kaczkowski B Podolska A Global microRNA Analysis of the NCI-60 Cancer Cell Panel Mol Cancer Ther 2011 10 375 384 10.1158/1535-7163.MCT-10-0605 21252286

[B35] Smyth GK Limma : linear models for microarray data Bioinform Comput Biol Solut using R Bioconductor 2005 397 420

[B36] Benjamini Y Hochberg Y Controlling the false discovery rate: a practical and powerful approach to multiple testing J R Stat Soc Ser B (Methodological) 1995 57 289 300

[B37] Matys V TRANSFAC(R): transcriptional regulation, from patterns to profiles Nucleic Acids Res 2003 31 374 378 [http://www.nar.oupjournals.org/cgi/doi/10.1093/nar/gkg108] 10.1093/nar/gkg108 12520026 PMC165555

[B38] Halees AS Weng Z PromoSer: improvements to the algorithm, visualization and accessibility Nucleic Acids Res 2004 32 Web Server issue W191 W194 [http://www.pubmedcentral.nih.gov/articlerender.fcgi?artid=441571&tool=pmcentrez&rendertype=abstract] 15215378 10.1093/nar/gkh433 PMC441571

[B39] Liu CC Lin CC Chen WSE Chen HY Chang PC Chen JJW Yang PC CRSD: a comprehensive web server for composite regulatory signature discovery Nucleic acids research 2006 34 Web Server issue W571 7 [http://www.ncbi.nlm.nih.gov/pubmed/16845073] 16845073 10.1093/nar/gkl279 PMC1538777

[B40] Griffiths-Jones S Grocock RJ van Dongen S Bateman A Enright AJ miRBase: microRNA sequences, targets and gene nomenclature Nucleic Acids Res 2006 34 Database issue D140 D144 [http://www.ncbi.nlm.nih.gov/pubmed/16381832] 16381832 10.1093/nar/gkj112 PMC1347474

[B41] Friedman N Linial M Using bayesian networks to analyze expression data J Comput Biol 2000 7 601 620 10.1089/106652700750050961 11108481

[B42] Liu B Li J Tsykin A Liu L Gaur AB Goodall GJ Exploring complex miRNA-mRNA regulatory networks by splitting-averaging strategy BMC Bioinformatics 2009 19 1 19 10.1186/1471-2105-10-408 PMC2797807 20003267

[B43] Chickering D Geiger D Heckerman D Learning Bayesian networks is NP-hard Technical Report MSR-TR-94-17, Vol. 196. Microsoft Research 1994

[B44] de Campos L A scoring function for learning bayesian networks based on mutual information and conditional independence tests J Machine Learn Res 2007 7 2 2149

[B45] Heckerman D Geiger D Chickering D Learning Bayesian networks: The combination of knowledge and statistical data Mach Learn 1995 20 3 197 243

[B46] Neapolitan R Learning Bayesian Networks 2003 Upper Saddle River: Prentice Hall

[B47] Murphy K The bayes net toolbox for matlab Comput Sci Stat 2001 33 2 1024 1034

[B48] Davidson A Hinkley D Bootstrap Methods and their Application 1997 Cambridge: Cambridge University Press

[B49] Peck R Devore J Statistics: The Exploration and Analysis of Data 1997 Pacific Grove: Duxbury Press

[B50] Audenaert P Van Parys T Brondel F Pickavet M Demeester P Van de Peer Y Michoel T CyClus3D: a Cytoscape plugin for clustering network motifs in integrated networks Bioinformatics (Oxford, England) 2011 27 11 1587 1588 [http://www.ncbi.nlm.nih.gov/pubmed/21478195] 10.1093/bioinformatics/btr182 21478195

[B51] Milo R Shen-Orr S Itzkovitz S Kashtan N Chklovskii D Alon U Network motifs: simple building blocks of complex networks Science 2002 298 824 827 10.1126/science.298.5594.824 12399590

[B52] Knabe JF Nehaniv CL Schilstra MJ Do motifs reflect evolved function? - No convergent evolution of genetic regulatory network subgraph topologies Biosystems 2008 68 74 18611431 10.1016/j.biosystems.2008.05.012

[B53] Gregory Pa Bert AG Paterson EL Barry SC Tsykin A Farshid G Vadas Ma Khew-Goodall Y Goodall GJ The miR-200 family and miR-205 regulate epithelial to mesenchymal transition by targeting ZEB1 and SIP1 Nat Cell Biol 2008 10 5 593 601 [http://www.ncbi.nlm.nih.gov/pubmed/18376396] 10.1038/ncb1722 18376396

[B54] Korpal M Lee ES Hu G Kang Y The miR-200 family inhibits epithelial-mesenchymal transition and cancer cell migration by direct targeting of E-cadherin transcriptional repressors ZEB1 and ZEB2 J Biol Chem 2008 283 22 14910 14914 [http://www.ncbi.nlm.nih.gov/pubmed/18411277] 10.1074/jbc.C800074200 18411277 PMC3258899

[B55] Zeisberg M Neilson EG Biomarkers for epithelial-mesenchymal transitions J Clin Invest 2009 119 6 1429 1437 10.1172/JCI36183 19487819 PMC2689132

[B56] Barrallo-Gimeno A Nieto MA The snail genes as inducers of cell movement and survival: implications in development and cancer Dev Suppl 2005 132 14 3151 3161 [http://www.ncbi.nlm.nih.gov/pubmed/15983400] 10.1242/dev.01907 15983400

[B57] Liu Y Yin J Abou-Kheir W Hynes P Casey O Fang L Yi M Stephens R Seng V Sheppard-Tillman H Martin P Kelly DR MiR-1 and miR-200 inhibit EMT via Slug-dependent and tumorigenesis via Slug-independent mechanisms Oncegene 2012 10.1038/onc.2012.58 PMC7580497 22370643

[B58] Shirakihara T Saitoh M Miyazono K Differential regulation of epithelial and mesenchymal markers by deltaEF1 proteins in Ephithelial-Mesenchymal transition induced by TGF-beta Mol Biol Cell 2007 18 3533 3544 10.1091/mbc.E07-03-0249 17615296 PMC1951739

[B59] Lee JM Dedhar S Kalluri R Thompson EW The epithelial-mesenchymal transition: new insights in signaling, development, and disease J Cell Biol 2006 172 7 973 981 [http://www.pubmedcentral.nih.gov/articlerender.fcgi?artid=2063755&tool=pmcentrez&rendertype=abstract] 10.1083/jcb.200601018 16567498 PMC2063755

[B60] Brabletz S Brabletz T The ZEB/miR-200 feedback loop—a motor of cellular plasticity in development and cancer? EMBO Reports 2010 11 9 670 677 10.1038/embor.2010.117 20706219 PMC2933868

